# Inflammatory signatures across four photon radiotherapy and proton radiotherapy: mechanisms, mitigation, and quality of life impact

**DOI:** 10.37349/etat.2025.1002334

**Published:** 2025-08-28

**Authors:** Yuting Sheng, Daniel M. Han, Mark R. Wakefield, Yujiang Fang

**Affiliations:** National Technical University of Athens (NTUA), Greece; ^1^Department of Microbiology, Immunology & Pathology, Des Moines University College of Osteopathic Medicine, West Des Moines, IA 50266, USA; ^2^Department of Surgery, University of Missouri School of Medicine, Columbia, MO 65212, USA; ^3^Ellis Fischel Cancer Center, University of Missouri School of Medicine, Columbia, MO 65212, USA

**Keywords:** Radiation-induced inflammation, radiation therapy modalities, quality of life, proton therapy

## Abstract

Cancer is the second leading cause of death globally and in the United States, second only to cardiovascular disease. Unlike many cardiovascular conditions, cancer is often less preventable, manageable, and curable—even with ongoing technological advancements in medicine. The adverse effects of cancer treatments on cancer patients remain profound due to shared cellular characteristics between cancerous and normal cells; one of the primary adverse effects is treatment-induced inflammation. These inflammatory responses aim to eliminate cancerous cells but often damage normal tissues. Notably, inflammatory side effects vary considerably across the growing diversity of therapeutic approaches. This study reviewed studies between 2007 and 2024, comparing the inflammatory profiles associated with five major radiation therapies (RTs): Three-Dimensional Conformal Radiation Therapy (3D-CRT), Intensity-Modulated Radiation Therapy (IMRT), Image-Guided Radiation Therapy (IGRT), Stereotactic Body Radiation Therapy (SBRT), and Proton Beam Therapy (PBT)—each characterized by distinct mechanistic and therapeutic features. In addition to each radiation modality eliciting distinct inflammatory responses, tissue-specific variability further complicates clinical outcomes. Accordingly, this review also undertakes a cross-tissue comparison of radiation-induced inflammation, with a focus on the gastrointestinal (GI) system, central nervous system (CNS), and skin. However, the variation in treatment modalities and organ-specific inflammatory biomarkers greatly hinders direct comparison across studies. Finally, this review highlights potential inflammatory mitigations, including ambroxol, that may be employed synergistically with RTs, minimizing side effects and enhancing patient outcomes. Taken together, while all modalities offer therapeutic value alongside certain limitations, proton-based therapy demonstrates the greatest potential for minimizing toxicity though its broader adoption remains limited by cost-effectiveness concerns.

## Introduction

Radiation therapy (RT) is one of the most prominent treatments for most cancer types, and currently, the primary modalities in RT are external radiation beams or brachytherapy. Both methods involve radioactive sources; however, brachytherapy, best for localized, easily accessible tumors, allows high doses of radiation to be delivered to a localized area by placing the radioactive source inside or very close to the tumor, minimizing the impact on surrounding tissue [1]. On the other hand, external radiation beams, widely applicable for many cancer types, utilize advanced techniques to direct a high-energy beam (photon or electron beams) at the tumor from outside the body; with a high degree of precision, external radiation beams effectively target and eradicate malignant tissues while limiting off-target effects on normal tissue [2].

XRTs (external beam RTs) such as 3D-CRT (Three-Dimensional Conformal Radiation Therapy) use advanced, computer-generated 3D imaging to precisely conform the radiation dose to the spatial geometry of the tumor. This approach enhances therapeutic efficacy while minimizing radiation exposure to adjacent normal tissues, thereby reducing potential off-target effects [3]. In addition to inducing tumor cell death via direct DNA damage, SBRT (Stereotactic Body Radiation Therapy) also exerts effects by disrupting tumor vasculature and exacerbating tumor hypoxia. These mechanisms contribute to secondary or indirect tumor cell death, amplifying its therapeutic impact [4]. Furthermore, the substantial release of tumor-associated antigens and various immunomodulatory factors, arising from both primary and secondary cell death, potentiates the activation of anticancer immune responses [5].

An immune response is intentionally induced in RT to target the tumor cells. Immunogenic cell death (ICD) is when irradiated tumor cells from RT release damage-associated molecular patterns (DAMPs) that induce inflammation and an immune response. The benefit of ICD is that the host’s immune system can attack the tumor cells not just at the irradiated site but also at distant sites using the CD8+ anti-cancer response. The types of DAMPs from the damaged tumor cells after RT include calreticulin (CALR), adenosine triphosphate (ATP), type I interferon (IFN), heat shock proteins (HSPs), S100 proteins, and interleukin-1 beta (IL-1β) [6]. DAMPs play a significant role in antigen-presenting, promoting inflammation, recruiting innate and adaptive immune cells, and promoting and activating CD8+ cytotoxic T cells [6].

**Figure 1:**
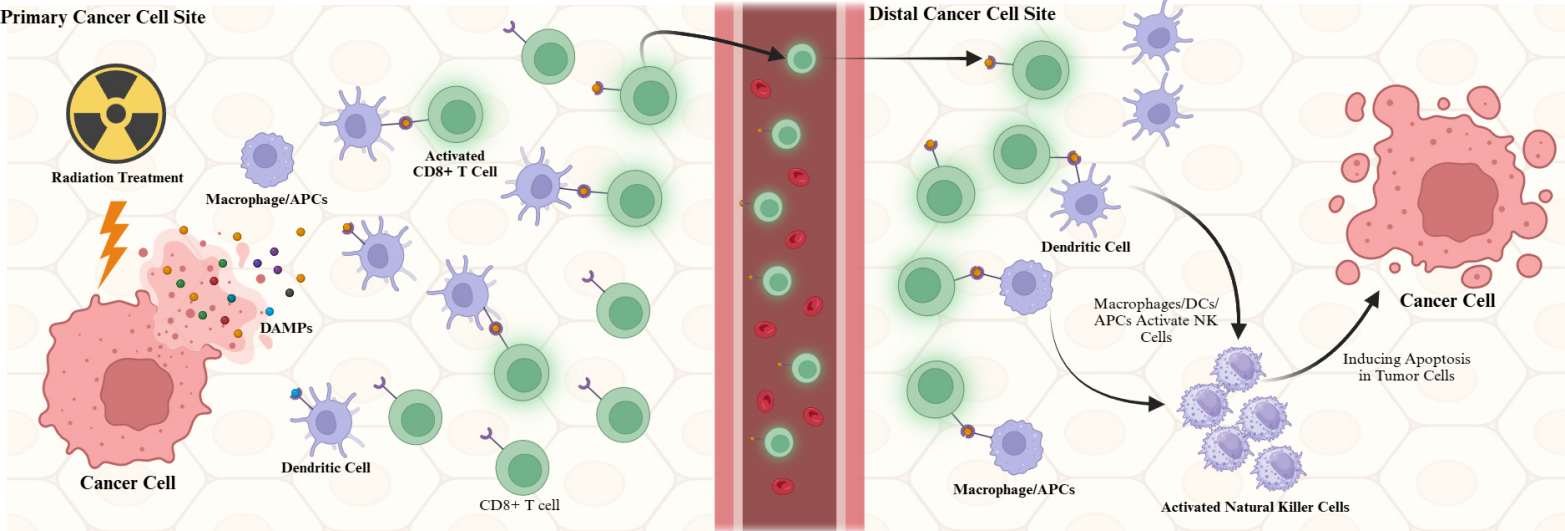
**Mechanism of radiation-induced immune activation and the abscopal effect in metastatic cancer.** Radiation therapy causes damage in cancer cells, and the damage-associated molecular patterns (DAMPs) released from the cancer cells are detected by dendritic cells (DCs) and antigen-presenting cells (APCs). These DAMPs bind DCs and APCs, travel to the distal cancer cells via circulation, and activate CD8+ T cells and natural killer (NK) cells to induce apoptosis in the distal tumor cells. Created in BioRender. Sheng, Y. (2025) https://BioRender.com/lvdqhb9

RT induces necrosis in targeted tumor cells. During necrosis, DAMPs, which could be damaged organelles, misfolded proteins, or DNA, will be released. DAMPs will trigger the innate immune response by activating pattern recognition receptors (PRRs) [7]. The antigen-presenting cells (APCs), such as dendritic cells (DCs), natural killer (NK) cells, neutrophils, and macrophages, will migrate to the irradiated cells and phagocytose the dead and dying tumor cells. The secondary or adaptive immune response happens when DCs phagocytose the antigens of the tumor cells and migrate to the lymph nodes. DCs will present the antigens to T cells, initiating adaptive immunity [8]. While innate immunity focuses on the site of irradiated tumor cells, adaptive immunity is at work to attack and regress tumor cells outside of the irradiated area. This phenomenon is called the abscopal effect (Figure 1) [9].

However, previous studies have also discovered that radiation-induced inflammation can harm immunocompromised patients.

Considering the profound effect RT has had on cancer and non-cancer diseases recently and the significant increase in cancer prevalence and incidence over the next 50 years, the objective of this review is to evaluate five of the most prominent and contemporary types of RT and patients’ responses to regulating each therapy’s toxicity, in particular, via patients’ inflammation and immune response [10–12].

## Radiation-induced inflammation and toxicity

Conversely, when normal tissues are unintentionally exposed to radiation during cancer therapy, the resulting immune activation can lead to sustained inflammation. Over time, this radiation-induced toxicity may contribute to progressive tissue damage, organ dysfunction, and a significant decline in the quality of life among cancer survivors [13]. The following subsections will explore the underlying mechanisms of radiation-induced inflammation and associated toxicity, supported by both experimental findings and clinical data. Additionally, emerging strategies for mitigating these adverse effects will be discussed, with an emphasis on improving patient outcomes and supporting individuals throughout the course of their radiation treatment.

### Mechanisms of radiation-induced inflammation

As with any inflammation process, RT-induced inflammation starts with acute inflammation and then progresses into chronic inflammation. Activated DCs induce inflammatory responses via cross-presenting tumor antigens to prime tumor-containing CD8+ T cells, polarizing immune cells into tumor suppression, or stimulating NK cells to sustain T-cell responses. However, if acute inflammation persists, it transitions into chronic inflammation; cancer cells not only hijack DCs to inhibit tumor antigen presentation, preventing innate and anti-tumor immunity, but also recruit extensively more immunosuppressive cells, providing a rich proangiogenic and pro-tumoral environment [14].

### Biomarkers of inflammatory toxicity

Inflammatory toxicity is a common adverse effect of RT, driven by both local tissue damage and systemic immune responses. As our understanding of radiation-induced inflammation deepens, a variety of circulating biomarkers have emerged as valuable tools for assessing the extent and severity of this toxicity, particularly due to their accessibility in peripheral blood. Among these, C-reactive protein (CRP) is a liver-derived acute-phase protein upregulated by proinflammatory cytokines such as IL-6, and reflects systemic inflammatory burden, while interleukin-1 receptor antagonist (IL-1Ra) acts as a natural inhibitor of IL-1 signaling, often elevated in response to excess proinflammatory cytokine activity. On the other hand, the neutrophil-to-lymphocyte ratio (NLR) captures the balance between innate immune activation and adaptive immune suppression, with elevated NLR indicating neutrophil-driven inflammation and reduced lymphocyte-mediated anti-tumor immunity. Similarly, yet distinctively, the monocyte-to-lymphocyte ratio (MLR) reflects chronic inflammation mediated by monocyte activation and a concurrent reduction in lymphocyte-driven anti-tumor immunity. Together, these biomarkers offer mechanistic insight into the inflammatory processes underlying radiation-related side effects, including fatigue, tissue injury, and poor clinical outcomes.

Previous studies have identified inflammatory biomarkers such as CRP and IL-1Ra as key downstream indicators of proinflammatory cytokine network activation. These markers not only reflect systemic inflammation but have also been significantly associated with fatigue during RT for breast and prostate cancers [15]. This association is likely due to the heightened release of proinflammatory cytokines—including IL-1β, IL-6, and TNF-α (tumor necrosis factor-alpha)—not only within the tumor microenvironment but also in adjacent healthy tissues, a consequence of the limited precision in conventional radiation techniques. These findings underscore the relevance of CRP and IL-1Ra as biologically meaningful indicators of radiation-induced inflammatory toxicity and their potential value in predicting therapy-related fatigue.

In addition, MLR has been identified as a potential indicator of radiation-induced toxicity and patient prognosis. In a study by Tanaka et al. [16], patients with elevated MLR exhibited a significantly lower 6-month overall survival (OS) rate (49.8%) compared to those with lower MLR values (79.8%). While this finding underscores the prognostic value of MLR, it is important to consider the study population—patients with stage IV lung cancer receiving palliative radiotherapy. The inherently aggressive nature of the disease and limited life expectancy in this group, though a sobering clinical reality, impose constraints on long-term biomarker analysis and mechanistic exploration. Although the study provides valuable insight into MLR as a prognostic tool in the palliative care setting, further investigations involving longer-term follow-up in more diverse patient populations may be necessary to fully elucidate the biological significance of MLR in radiation-induced inflammatory toxicity.

Interestingly, a study by Takaoka et al. [17] investigating helical tomotherapy for liver metastases originating from pancreatic cancer has underscored the potential utility of inflammatory biomarkers as prospective prognostic indicators. The authors found that low pretreatment levels of NLR and MLR were paradoxically associated with poorer clinical outcomes, whereas lower MLR was otherwise typically correlated with improved OS rates in other contexts. This finding further reinforces the prognostic relevance of immune-based markers such as NLR and MLR in understanding cancer progression and guiding therapeutic decision-making.

Collectively, inflammatory biomarkers such as CRP, IL-1Ra, NLR, and MLR offer a valuable snapshot of the dynamic interplay between pro- and anti-inflammatory forces during RT. Elevated levels may reflect an exaggerated immune response involving both malignant and surrounding healthy tissues, contributing to adverse outcomes such as fatigue, reduced survival, and overall poor prognosis. The routine monitoring of these biomarkers could facilitate early detection of radiation-induced toxicity and inform personalized treatment strategies. Nonetheless, numerous questions remain in this field—particularly regarding the optimal patient populations for long-term follow-up. Carefully designed prospective studies with extended observation periods will be essential for bridging existing knowledge gaps. Importantly, future research integrating these hematologic biomarkers with cytokine profiling may further elucidate their predictive utility and support the development of inflammation-targeted interventions in radiotherapy.

### Acute versus chronic inflammatory toxicities

Previous studies have discovered that RT can induce ICD of irradiated tumor cells. DAMPs such as proteins, nucleic acids, and metabolites leak from damaged, dying cells. DCs and other APCs are activated by binding to these DAMPs. After APC and DC activation, they mediate anti-tumor T-cell responses [18]. Additionally, the innate immune response plays a significant role in self-amplified anti-tumor response. Activated anti-tumor T-cells will migrate to the irradiated site, inducing apoptosis in the damaged tumor cells. This will result in increased DAMPs concentration due to more tumor cells being damaged and dying. These DAMPs will induce more DCs and APCs at the site, increasing the immune response [19]. Adaptive immune response mediated by T-cells prevents metastasis and induces anti-tumor immunity by killing distant tumor cells after developing anti-tumor immunity [9].

Although previous studies have shown the benefits of having an innate immune response after RT through ICD, Wu et al. [20] demonstrated that radiation can induce inflammation through the IL-6/STAT3 signaling pathway, helping tumor cells facilitate invasion and survival after therapy. Additionally, studies by Culig and Puhr [21] have shown that silencing IL-6 using siRNA prevents tumor recurrence following radiotherapy in prostate cancer and increases tumor cells’ sensitivity to radiation.

Chronic inflammation is known as one of the key players in cancer development [22]. Previous studies done by Greten and Grivennikov [23] in 2019 demonstrated that cancer-associated genes recruit and activate pro-inflammatory cells, leading to tumor progression. Browning et al. [24] in 2018 showed that inflammation induces pro-inflammatory IL-6, which then activates the JAK/STAT3 pathway for malignant tumor proliferation. Studies done by Theoharides [25] in 2008 demonstrated that the oncogenic *MYC* gene induces the recruitment of mast cells, which are responsible for angiogenesis and expansion to tumor sites.

**Figure 2:**
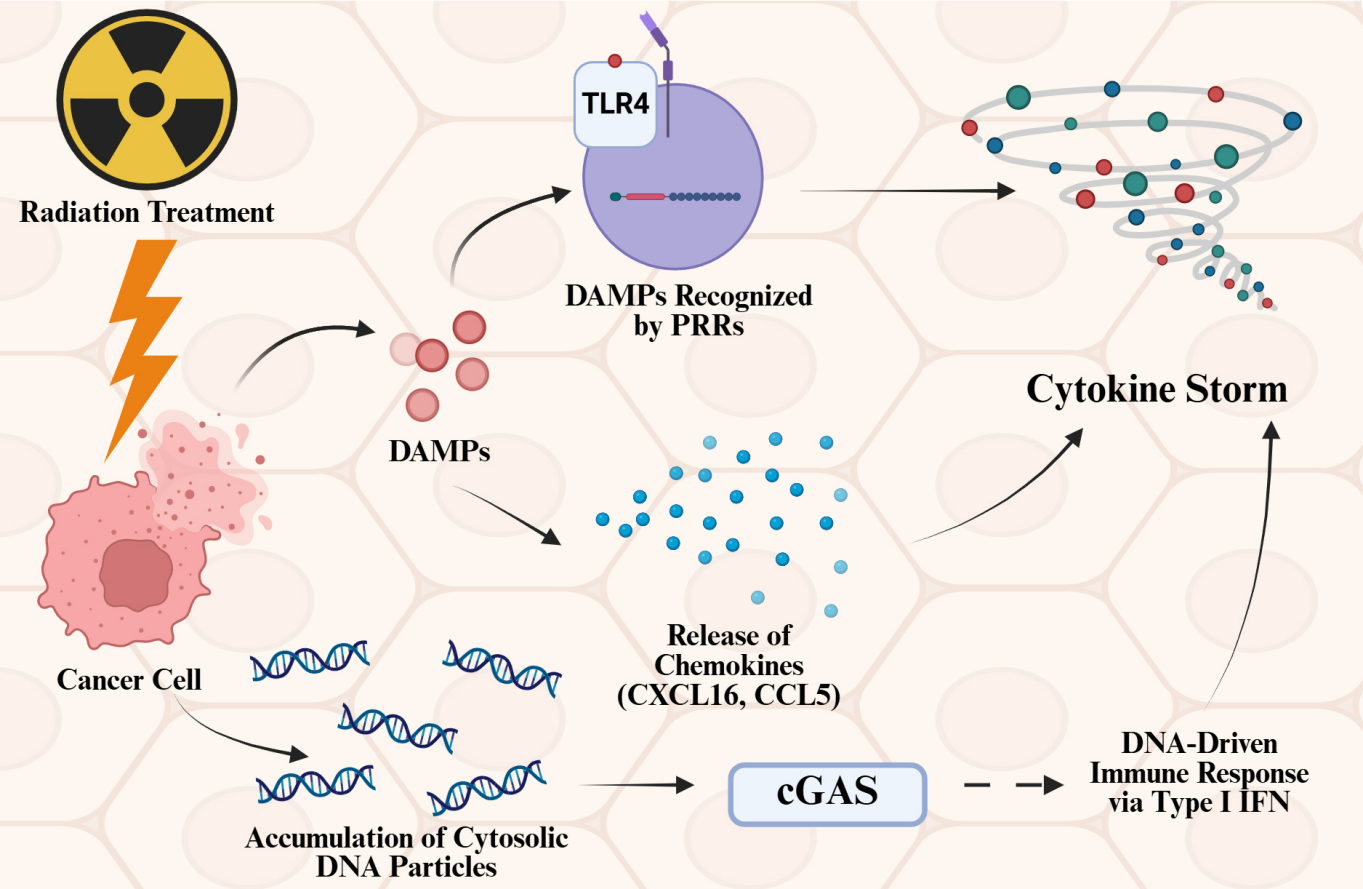
**Mechanisms of radiation-induced cytokine storm and inflammation.** Radiation treatment can trigger cytokine storms via 3 different pathways. Radiation treatment induces damage in cancer cells, and damage-associated molecular patterns (DAMPs) released from the cancer cells are sensitized by pattern recognition receptors (PRRs), leading to cytokine storms. These DAMPs can also induce the release of chemokines such as CXCL16 and CCL5, which lead to cytokine storms. When nuclei of cancer cells are damaged, DNA particles can accumulate in the cytosol, and these DNA particles can activate the cGAS pathway, which will induce a DNA-driven immune response via type I interferon (IFN) and ultimately induce cytokine storms. Created in BioRender. Sheng, Y. (2025) https://BioRender.com/x03tud5

Furthermore, previous studies have shown that RT can reinforce the harmful side effects of inflammation. Zhang et al. [26] demonstrated that RT can potentially induce a cytokine storm (Figure 2). The three pathways they demonstrated are: (1) pro-inflammatory responses initiated by DAMPs interacting with PRRs; (2) the release of chemokines like CXCL16 and CCL5; and (3) the activation of neoantigen presentation alongside the cGAS-STING signaling pathway. Previous studies, done by Schaue et al. [27], have demonstrated that radiation affects both acute and chronic inflammatory disease.

### Organ-specific inflammatory responses

Different organs respond differently to RT due to variations in their cell types and immune response programming. Evidence suggests that RT-induced inflammation activates endothelial cells to produce inflammatory mediators. As a result, the CD11/CD18 on leukocytes is up-regulated, increasing NF-κB-dependent endothelial cell adhesion molecule (CAM) expression. This will assist the leukocyte adhesion and reinforce the extravasation of inflammatory cells into the GI (gastrointestinal) tissues, thus inducing organ damage [28].

Unlike the GI tract’s reliance on endothelial cells, the CNS (central nervous system) depends on microglia to detect and respond to radiation. Upon ionizing radiation (IR), microglia swiftly engage neurons, astrocytes, and oligodendrocytes to orchestrate CNS-specific defenses—modulating the blood-brain barrier (BBB), controlling peripheral immune cell entry, and inhibiting neurogenesis [29]. Although IR universally activates DNA damage pathways (NF-κB, CREB, AP1) and upregulates IL-1β, TNF-α, COX2, and MCP1, the CNS’s inflammatory response is uniquely gated by microglial expression of tight junction proteins like claudin5, which more stringently limits peripheral infiltration than in other tissues [30]. Radiation also disrupts the anatomical support between neural progenitors and their microvasculature; the ensuing microglial inflammation alone suffices to sever neurovascular contacts and suppress stem cell function [31]. Coupled with the brain’s inherently poor regenerative capacity, these effects explain the progressive deficits in memory, attention, and executive function observed months to years after cranial irradiation [32].

Skin is one of the most common sites to experience radiation-induced damage after RT. Previous studies have revealed that 95% of the patients who were treated with RT experienced skin changes [33]. In part of the skin’s innate response, Langerhans cells reside in the epidermal layer, and DCs and resident T cells reside in the dermal layer. They have a significant role in initiating immune responses against barrier injury. Antigen-specific effector T cells, such as CD4+, CD8+, and Treg, are responsible for adaptive immunity. There are also unconventional lymphocyte subsets, such as αβ and γδ T cells, which are considered part of innate immunity [34]. Radiation dermatitis refers to skin inflammation resulting from exposure to IR, commonly occurring as a side effect of RT or, less frequently, due to accidental nuclear exposure. Studies have shown that radiation-induced skin damage is driven by IL-17—producing γδ T cells and innate lymphoid cells. This process involves increased expression of IL-1β and IL-23 at the site of injury, which subsequently promotes keratinocyte hyperproliferation [35]. This hyperproliferation of the keratinocytes will induce an imbalance in the keratinocytes in the skin layer, which ultimately contributes to skin damage.

While resident DCs and unconventional lymphocytes like γδ T cells play a significant role in immune response to RT in the skin system, the respiratory system relies heavily on innate immune cells, particularly macrophages. Previous studies on mice demonstrated that radiation damage activates cGAS-STING signaling through CCL2 and induces the M1 polarization of microphages. The M1 macrophages secrete proinflammatory mediators such as cytokines, ILs, and IFNs, ultimately leading to the development of radiation pneumonitis [36].

### Comparative analysis of inflammatory responses across radiation therapy modalities

Modern photon-based RTs triumph in their increasingly refined precision, which directly correlates with reduced inflammation and toxicity—two critical determinants of both patient tolerability and survival.

For instance, SBRT demonstrates high precision coupled with high-dose delivery over a limited number of fractions. In the study by Van’t Land et al. [37], the systemic immune-inflammation index (SIII)—a composite biomarker derived from neutrophil, platelet, and lymphocyte counts—was used to assess immune response. While no significant group-wide change in SIII was observed post-SBRT, patients who experienced a reduction in SIII demonstrated markedly improved OS (24.0 vs. 11.6 months; *P* < 0.036) [37]. This suggests that SBRT’s precision may limit unintended immune activation, and SIII may serve as a useful prognostic indicator to guide candidate selection and predict response.

By contrast, 3D-CRT—an earlier-generation photon-based modality—relies on static beam shaping and lacks the adaptive modulation seen in techniques like SBRT. Theoretically, its fixed geometry and broader treatment margins render it less precise than SBRT, potentially exposing more surrounding healthy tissues to radiation and increasing the risk of inflammatory toxicity. However, recent findings suggest a more nuanced profile: in a study on advanced bladder cancer, 3D-CRT combined with chemotherapy resulted in significantly lower post-treatment IL-6 levels than chemotherapy alone (63.43 ± 7.95 vs. 132.75 ± 16.12 ng/L), indicating a capacity to attenuate systemic inflammation [38]. More importantly, this anti-inflammatory effect translated into improved survival outcomes: the 1-year survival rate in the 3D-CRT combination group was 76.19% compared to 47.62% in the chemotherapy-only group, and the 2-year survival rate was 47.62% vs. 19.05%, respectively [38].

This outcome highlights a potential therapeutic synergy. While 3D-CRT alone may theoretically induce more collateral damage than other precision modalities such as IMRT (Intensity-Modulated Radiation Therapy), IGRT (Image-Guided Radiation Therapy), SBRT, or even Proton Beam Therapy (PBT), its role alongside chemotherapy appears to blunt harmful cytokine surges. Unlike the other therapies typically used as standalone treatments, 3D-CRT may derive greater utility when employed as a supportive adjunct—leveraging its spatial coverage to enhance tumor response, while relying on systemic agents to help control treatment-induced inflammation.

IMRT represents a significant leap in conformal dosing, enabling selective targeting even in anatomically complex regions. In a prospective analysis of patients with advanced non-small-cell lung cancer, IMRT significantly reduced the incidence of grade ≥ 3 pneumonitis compared to 3D-CRT (3.5% vs. 7.9%; *P* = 0.039), despite being applied to patients with larger and more advanced tumors [39]. This reduction in treatment-induced lung inflammation highlights the clinical significance of IMRT’s superior spatial precision, which allows for precise sculpting of dose away from critical normal lung tissue, thereby minimizing collateral damage. This finding underscores IMRT’s unique clinical utility in managing complex tumor presentations, particularly those located near critical structures such as the heart, esophagus, or major airways. While IMRT and SBRT share similar beam-shaping and image-guidance technologies, IMRT holds a distinct advantage in treating irregularly shaped or voluminous tumors. This is likely due to its ability to gradually deliver radiation over a larger number of fractions, allowing for effective dose distribution with reduced risk to surrounding normal tissues. In contrast, SBRT’s strength lies in its ultra-high-dose, high-precision delivery, which, although extremely effective for small, well-defined lesions, may not be suitable for larger or anatomically complex targets. The IMRT data in advanced lung cancer thus not only support the principle that greater spatial precision reduces toxicity but also illustrate the importance of fractionation flexibility in balancing tumor control and normal tissue preservation.

Last but not least, IGRT builds upon IMRT by integrating real-time imaging corrections to account for inter- and intrafractional motion. In a meta-analysis involving over 5,000 prostate cancer patients, IGRT was associated with significantly reduced rates of acute and late toxicity across multiple systems—GI [RR (relative risk) = 0.55, *P* = 0.007], GU (genitourinary) (RR = 0.82, *P* = 0.006), and late GI complications [HR (hazard ratio) = 0.25, *P* = 0.03] [40]. The data imply that IGRT’s precision extends beyond planning to execution, minimizing healthy tissue exposure and allowing for tighter margins without compromising safety.

In summary, the progression from 3D-CRT to IMRT, SBRT, and IGRT illustrates a clear trajectory: as spatial and temporal precision improve, inflammation-induced toxicity tends to decline, leading to improved patient outcomes and greater treatment tolerability. A common misconception, however, is the treatment of IGRT as a standalone radiation modality. In reality, IGRT serves as a powerful adjunctive technology whose primary function is to enhance the precision of other radiotherapy modalities by enabling real-time imaging and positional verification during treatment delivery.

Notably, the integration of IGRT benefits different modalities to varying degrees. While its impact on 3D-CRT is relatively limited—due to the broader treatment fields and static beam shaping inherent to that technique—its combination with IMRT and SBRT significantly amplifies their precision and safety profiles. This further reinforces why 3D-CRT, though once standard, is increasingly considered less favorable or even obsolete, not only for its limited responsiveness to IGRT enhancement, but also due to its higher risk of damaging healthy tissues. This further reinforces why 3D-CRT, though once standard, is increasingly considered less favorable or even obsolete, not only for its limited responsiveness to IGRT enhancement, but also due to its higher risk of damaging healthy tissues. However, it is important to note that the perceived precision hierarchy among IMRT, SBRT, and IGRT should not be oversimplified. While the overall trend reflects advancements in targeting accuracy, IMRT and SBRT each serve distinct roles based on tumor geometry, location, and clinical goals. SBRT offers unparalleled precision for small, well-defined lesions, whereas IMRT excels in conformal treatment of irregular or anatomically complex tumors. In this context, precision must be evaluated alongside tumor-specific characteristics rather than ranked in isolation. Importantly, precision alone does not determine clinical suitability; the anatomical complexity, size, and shape of the tumor often dictate whether IMRT or SBRT is more appropriate. Yet in both cases, the addition of IGRT confers substantial advantages by reducing setup uncertainties and enabling tighter treatment margins.

Even though this review has primarily examined photon-based modalities as standalone treatments, the observed benefits of integrating IGRT raise a critical question: Could the addition of IGRT to proton therapy similarly enhance its therapeutic precision by incorporating real-time image guidance? This possibility sets the stage for the emerging role of proton therapy as a highly precise modality and explores how it compares—both in targeting accuracy and immune response profiles—to its photon-based counterparts.

Photon-based therapies like SBRT, 3D-CRT, IMRT, or IGRT have revolutionized cancer treatment, but they still pose a challenge due to the unintended radiation exposure to nearby healthy tissues. In contrast, proton therapy stands out with its remarkable precision, reducing collateral damage and presenting a compelling alternative. A study by Li et al. [41] explored the effects of stereotactic body proton therapy (SBPT) compared to SBRT. Interestingly, SBPT cases showed a more rapid inflammatory response in the lungs than SBRT. While this heightened inflammatory reaction doesn’t appear to cause additional cellular toxicity or negatively affect patient outcomes, it could unlock exciting opportunities for synergy with immunotherapies, such as immune checkpoint inhibitors (ICIs). This accelerated immune response might offer a therapeutic edge in combining SBPT with ICI-based treatments.

Similarly, a study done by Davuluri et al. [42] has also shown the benefit of PBT compared to IMRT in esophageal cancer patients. This study focused on the measurement of lymphocyte count and divided it into 4 grades, 4 being the worst. The importance of immunity and lymphocytes has made lymphopenia a crucial topic when studying different cancer treatments. The study has shown a significant proportional association between mean body dosage (MBD) and grade 4 absolute lymphocyte count (G4 ALC) nadir. Likewise, patients with G4 ALC nadir had a higher MBD than those without (12.7 Gy vs. 10.8 Gy, *P* < 0.001). At last, the discovered patients treated with PBT had a significantly lower MBD (8.0 Gy vs. 13.2 Gy, *P* < 0.001) and a higher likelihood of having an MBD < 10 Gy (88.6% vs. 11.4%, *P* < 0.001) than did patients treated with IMRT concluded the benefits of proton ration therapies do not only reside in the lower MBD but more importantly in its protection toward host’s immunity.

### Mitigation and management strategies

Although normal tissue toxicity remains a major dose-limiting factor in RT and a significant contributor to long-term complications in cancer survivors, effective treatment options to mitigate these side effects are still limited. Currently, anti-inflammatory drugs are among the most commonly used interventions. At the same time, ongoing research is exploring novel therapeutic strategies to counteract radiation-induced toxicities. These approaches focus on reducing RT-induced apoptosis through various pathways, restoring post-radiation redox balance, and directly targeting inflammatory and fibrogenic signaling cascades [43].

When it comes to apoptosis, we cannot avoid talking about p53. In a study investigating the inhibition of p53-dependent apoptosis, Wang et al. [44] identified PUMA as a key p53 target that mediates radiation-induced apoptosis through a cell-intrinsic mechanism. Their findings further highlight CHIR99021, a GSK-3 inhibitor, as a potent radioprotective agent, offering a potential strategy to mitigate radiation-induced cellular damage. Although Wang et al. [44] could not directly assess radiation responses in human subjects, they examined the effects of CHIR99021 in human intestinal cultures derived from two unrelated donors. These cultures contained all differentiated epithelial lineages, providing a relevant model for studying intestinal regeneration. Their findings were promising: enteroid formation significantly improved 10 days after exposure to 5 Gy radiation in cultures treated with CHIR99021. Additionally, blocking PUMA induction led to a notable increase in the expression of key intestinal stem cell (ISC) markers, including *Olfm4*, *Lgr5*, *Ascl2*, and *CD44*. These molecular changes were accompanied by a significant reduction in apoptosis, decreased DNA damage, and enhanced cell proliferation, highlighting CHIR99021’s potential as a radioprotective agent.

In addition, free radicals are another main reason for cell damage, even in healthy individuals. Therefore, modulating these free radicals to restore redox equilibrium to reverse normal tissue injury is another critical mitigation for post-radiation patients. A study done by Mahmood et al. [45] discovered the benefit of a genistein diet toward radiation-induced oxidative damage in rats. They utilized the levels of 8-hydroxy-2-deoxyguanosine (8-OHdG), a marker of oxidative damage in the DNA, and levels of malondialdehyde (MDA), a measure of lipid peroxidation. As expected, the treatment group showed a decreasing trend in 8-OHdG, and MDA concentration was also significantly decreased by the genistein diet. Furthermore, a higher survival rate of irradiated rats was associated with the treatment group, where a proportion of the control group had to be euthanized due to excessive morbidity between 6–18 weeks; however, almost all rats in the treatment group survived to the end of the study at 36 weeks. Even though genistein is only one substance dedicated to restoring redox equilibrium, it also sheds light on the inflammatory and fibrogenic pathways contributing to tissue damage post-RT, supporting research in direct signal intervention targeting inflammatory and fibrogenic signals.

Lastly, an effective substance, ambroxol, is a great protective agent against the transforming growth factor beta 1 (TGF-β1) and TNF-α, studied by Xia et al. [46]. TGF-β1 is believed to have the closest relationship with the generation of radiation lung fibrosis, significantly diminishing patients’ quality of life. The study showed a significant decrease in the level of TGF-β1 in the treatment group compared to the control. In addition, ambroxol was also observed to significantly abolish the production of post-radiation TNF-α. The dual effect, on early-phase radiation pneumonitis (TNF-α) and late-phase lung fibrosis (TGF-β1), makes ambroxol a novel supplement for RT patients.

### Impact on quality of life and survivorship

In a recent comparative study of proton radiotherapy (PT) versus photon therapy, Baumann et al. [47] found no significant difference in disease-free or OS between the two modalities. However, they observed a two-thirds reduction in adverse events associated with unplanned hospitalizations of the proton chemoradiotherapy cohort, even though that cohort was significantly older and had less favorable Charlson-Deyo comorbidity scores.

Similarly, a prospective randomized phase II trial presented its favorable preliminary results for PT when compared to IMRT on glioblastoma. Although there was no significant difference in time to cognitive failure, progression-free survival (PFS), or OS, the PT cohort reported a lower rate of fatigue at 24% compared to 58% for the IMRT population. Furthermore, PT showed a significant reduction in the radiation dose in all structures; as a result, it only showed a mean of 0.35 grade of between 0–3 grade toxicities. On the other hand, IMRT had a range of grade 0–6 toxicities and an average number of 1.15, which is significantly higher in grade toxicity and the number of them [48].

In another randomized phase IIB trial carried out by Lin et al. [49], PT and IMRT were compared again for locally advanced esophageal cancer. In agreement with the previous trial, a total toxicity burden (TTB) was calculated to be 2.3 times higher in the IMRT group than the PT, and similar 3-year PFS rate and OS rates were observed in IMRT and PT to be 44.5% vs. 44.5% and 50.8% vs. 51.2%, respectively. Once again, a lower toxicity is observed in the proton cohort; however, no increase in survival rate has been observed due to the significant difference in toxicity.

Lastly, de González et al. [50] are working on the first large-scale systematic comparison between proton and photon therapy in a variety of pediatric cancers that are commonly treated with radiation from 2007 to 2022. This comparison between the two modalities and the analysis of subsequent cancer risk is an effort to shine light on the clinical practice of pediatric patients, but also hopefully transcend beyond the pediatric cohort, into the lives of everyone else.

Whether it is glioblastoma or advanced esophageal cancer, cohorts showed a better tolerance toward proton therapy, even though the OS rate has consistently shown no significant difference. Such an effect might be explained by the mechanisms and pathways each modality affects. And we must combine their fundamentalities with the clinical data explained above to comprehensively assess which therapy would be more beneficial to cancer cells, but also to patients themselves.

One of the most significant distinctions between proton and photon therapies is the mechanism responsible for proton therapy’s low toxicity—the Bragg peak—which is considered a cornerstone of proton therapy. This unique physical characteristic allows particle therapy to precisely deliver calculated radiation doses to the tumor while effectively sparing surrounding healthy tissues. The advantage lies in proton therapy’s superior ability to preserve normal tissues while providing radiation doses equivalent to, or even greater than, those administered by photon therapy. According to Byun et al. [51], charged particle beams exhibit a distinct depth-dose distribution as they traverse biological tissues towards cancerous lesions; specifically, a steep, localized peak of radiation occurs when particles deposit the majority of their energy in the last few millimeters of their path as they decelerate. This delayed yet concentrated energy deposition enables protons to effectively target abnormal tissues without prematurely releasing radiation energy into healthy structures, thereby maximizing therapeutic efficacy while minimizing toxicity.

In contrast, photons immediately release most of their dose upon their entry and gradually decrease as a function of distance. As a result, there will not only be a dampened effect on cancerous tissues, but also there is a significantly greater toxicity experienced by healthy tissues [50]. However, after the arrival of radiation dosage, both modalities will induce similar biological mechanisms at the cellular level—DNA damage and G2/M arrest, inhibition of cell proliferation, and controlled and quantifiable apoptosis [52].

## Discussion

The role of inflammation and immune response in RT has become a critical area of investigation, particularly as our understanding of tumor microenvironments and immune modulation evolves. This review explored the complex interplay between radiation-induced inflammation and immune system activation, highlighting how various RT modalities, including 3D-CRT, IMRT, IGRT, SRT, and PBT, influence these biological processes. While RT primarily targets tumor cells, its impact on surrounding normal tissues and the immune response has gained increasing attention as a key determinant of treatment success and therapeutic enhancement.

Notably, radiation-induced inflammation can cause significant damage to cancer patients, further compromising their overall health. Among the studies we reviewed, PBT appears to be a less harmful treatment option. However, as noted in previous sections, there are ongoing investigations into various mitigation strategies, including the use of ambroxol. Its promising effects should be further explored to protect the health of cancer patients undergoing RT, potentially improving survival rates.

**Figure 3:**
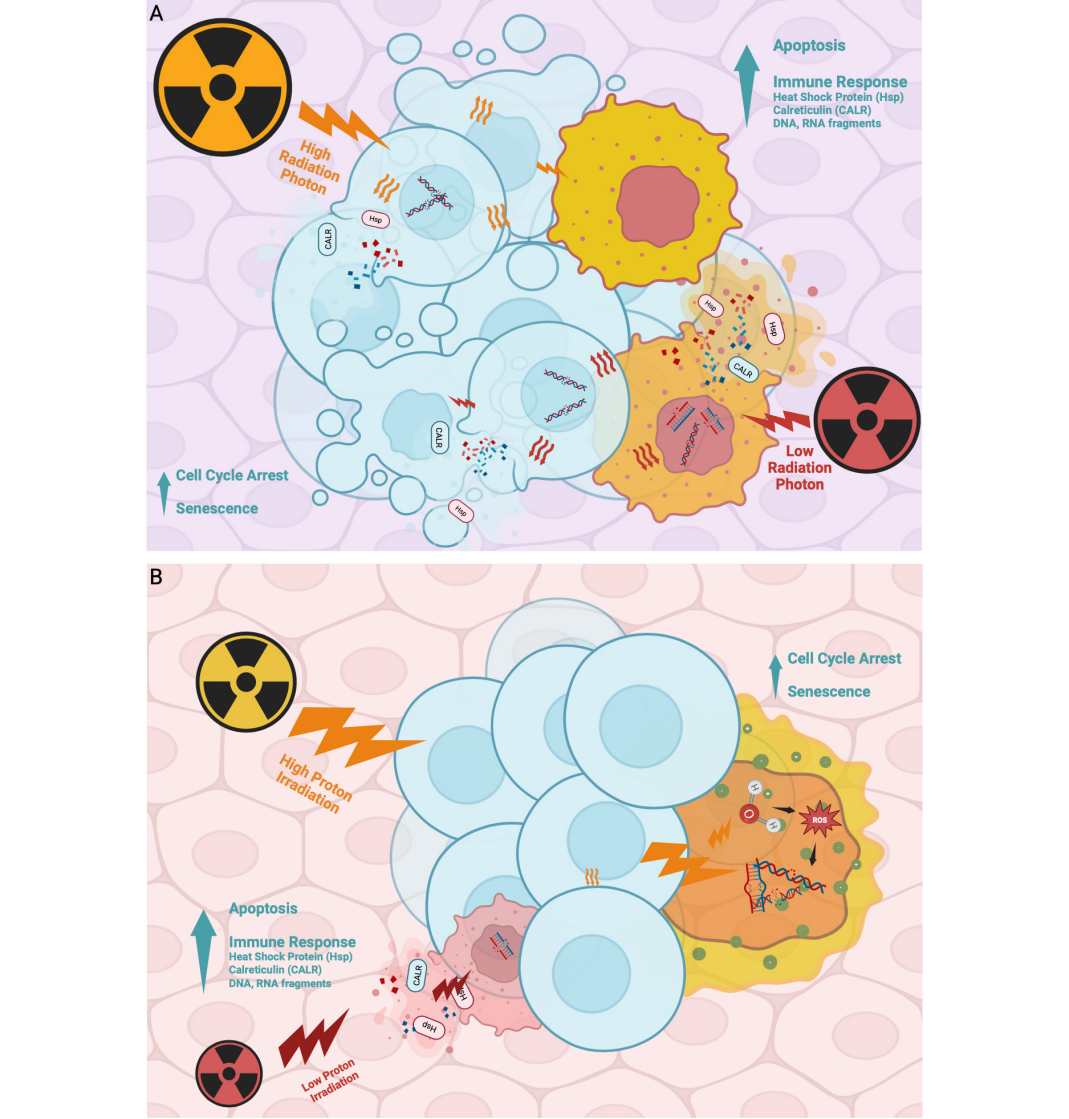
**Comparative effects of photon and proton radiation on tumor cells and tissue penetration.** (**A**) Photon radiation rapidly dissipates energy as it enters tissue, delivering most of the dose to superficial layers regardless of energy level. This limits its effectiveness for treating deep-seated tumors, as insufficient energy reaches the tumor site. Additionally, healthy tissues along the beam path absorb much of the radiation, increasing the risk of collateral damage. (**B**) Proton radiation induces comparable tumor cell responses—including apoptosis, senescence, and immune signaling—but features a unique physical property known as the Bragg peak. By adjusting beam energy, clinicians can concentrate radiation at a specific tissue depth with minimal entry and exit dose. This enables greater precision, reduced damage to healthy tissue, and more effective treatment of tumors located deeper in the body. Created in BioRender. Sheng, Y. (2025) https://BioRender.com/8vryot1

One of the principal reasons photon RT induces substantial collateral damage lies in its underlying dose-distribution mechanism. Photon beams exhibit an exponential dose fall-off with depth, delivering substantial energy to all tissues along their path and lacking a comparable mechanism for concentrated deposition at a specific depth. In contrast, photon radiation dissipates rapidly upon entering tissue, depositing most of its energy in superficial layers; while effective for surface-level tumors, this limits its efficacy against deep-seated malignancies, as insufficient energy reaches tumor cells at depth to elicit robust cytotoxic or immune-mediated responses (Figure 3A).

Although photon and proton therapies share fundamental biological mechanisms, proton beam depth-dose modulation enables precise control over penetration: higher-energy protons travel farther through tissue with minimal energy loss along their path, conserving dose for maximal deposition at the deep tumor site; conversely, lower-energy protons deposit most of their energy more superficially, limiting penetration beyond superficial malignancies. This dose-dependent, depth-varying energy release—termed the Bragg peak—permits high-precision targeting of tumor volumes while minimizing exposure to both intervening and downstream healthy structures. The hallmark Bragg peak of proton beams confers superior dose conformity—maximizing tumoricidal effects while sparing surrounding healthy tissue (Figure 3B).

Despite proton therapy’s dosimetric advantages, it has not demonstrated a clear OS benefit, and its substantial cost can easily outweigh these subtle clinical gains [53]. A comprehensive analysis by Doyen et al. [54] across eleven cancer types—from pancreatic adenocarcinoma to pleural mesothelioma and Hodgkin lymphoma to prostate adenocarcinoma—arrived at a similar conclusion: the theoretical benefits of proton therapy may not justify its routine use in all patients, yet should be prioritized in younger individuals and those with longer expected survival. This is particularly true for conditions such as Hodgkin lymphoma and breast cancer, where proton therapy’s reduced cardiac toxicity and lower risk of radiation-induced secondary malignancies translate into meaningful improvements in long-term quality of life.

PBT yielded the most favorable clinical outcomes in our review; however, its relative novelty, markedly higher costs compared with conventional modalities, and restrictive insurance coverage—evidenced by a 63 percent initial denial rate—have limited its availability [55]. Interestingly, the real reason why PBT is so costly is due to its astronomical upfront cost; first-generation proton therapy facilities require the construction of entirely new buildings with ample space to accommodate large, multi-room machines and the substantial concrete shielding necessary for safe operation. Historically, conventional multiroom proton therapy facilities—typically equipped with four to five treatment suites—have required capital investments ranging from $100 million to $300 million, with annual operational expenditures estimated between $15 million and $25 million [56]. However, recent technological advancements have significantly illuminated the potential to reduce these exorbitant expenses. The shift from multi-room to single-room proton machines has dramatically lowered the cost of establishing a new proton center to approximately $30 million—an order of magnitude less than previous models [57]. Importantly, these compact single-room systems maintain high levels of accuracy and reproducibility when compared with conventional multiroom technology [58]. Additionally, the reduced physical footprint of compact proton therapy systems facilitates their integration into existing cancer centers, eliminating the need for standalone facilities in major metropolitan areas. This advancement not only lowers the threshold for patient volume required to sustain operations but also creates the potential for implementation in already established rural hospitals, thereby expanding access to underserved regions. This can be made increasingly feasible through governmental grants or private investment, making proton therapy more financially accessible to patients across diverse socioeconomic and geographic backgrounds. These avenues of support should be actively considered as part of the strategic development and future direction in the domain of proton therapy.

These promising developments continue to enhance the clinical appeal of proton therapy, positioning it as a potential mainstream, cutting-edge cancer treatment. However, emerging research has raised concerns regarding the distinct biological effects of proton therapy’s IR, which may diminish its clinical advantages over conventional photon RT. A review by Mavragani et al. [59] examined the impact of IR on complex DNA damage and reported that protons, particularly at high linear energy transfer (LET) levels (e.g., ~ 10 keV/μm), not only induce conventional DNA lesions such as single-strand breaks and base lesions but also generate clustered, spatiotemporally localized damage involving multiple lesions. These complex DNA damages are more difficult to repair, as evidenced by a slower repair rate of double-strand breaks (DSBs) induced by high-LET radiation. While proton therapy allows for more precise tumor targeting, its biological effects—though spatially confined—are characterized by greater complexity and delayed repair, potentially resulting in more severe cellular consequences than the diffuse yet simpler DNA damage typically associated with photon therapy.

Nevertheless, a direct comparison between the cumulative but simpler DNA damage from photon therapy and the more localized, complex damage from proton therapy is not straightforward, as cellular response varies widely by cancer type and individual patient factors. This highlights the need for more case-specific evaluations and expanded future research in this area. Notably, studies have shown that proton therapy induces a higher frequency of DSBs and micronuclei formation compared to photon therapy at equivalent doses (2–12 Gy) [60]. These DSBs initiate a cascade of DNA damage responses, including micronuclei formation, which in turn activates innate and adaptive immune pathways [61, 62]. For instance, a single 16.4 Gy dose of proton radiation was found to activate type I IFN signaling and enhance cytolytic activity via antigen recognition, as demonstrated by Mirjolet et al. [63]. Thus, although the complex DNA damage caused by proton therapy may appear detrimental, it also contributes to enhanced immune activation, making its biological mechanisms comparable in benefit to those of photon therapy. This underscores the difficulty of characterizing proton therapy solely by its DNA damage profile. Despite the variability in findings across studies, a consistent theme is the urgent need for further investigation into the unique mechanisms of proton-induced DNA damage and their therapeutic implications, particularly in relation to immune modulation. Future studies employing advanced analytical tools may offer more comprehensive insights into this complex interplay.

Given that proton therapy remains an evolving modality with many of its biological mechanisms and long-term clinical benefits still being studied, it is challenging to rely solely on current clinical data to guide its implementation. Nevertheless, establishing contemporary parameters for patient selection—grounded in existing evidence and mechanistic insights—can help optimize its judicious use in appropriate populations, while also minimizing the risks associated with effects still under investigation and reducing the financial burden placed on patients. In other words, patient selection should be guided by a careful evaluation of both medical necessity and socioeconomic feasibility. For example, a recent study by Nantavithya et al. [64] found that although PBT is substantially more expensive than XRT, it is more effective in treating medulloblastoma due to its reduced neurocognitive side effects. The study highlighted that the IQ (intelligence quotient) decline associated with XRT can lead to downstream opportunity costs in education and employment, particularly in younger patients. Accordingly, the researchers emphasized the need for future studies to guide PBT utilization based on patient demographics. Younger patients, who stand to gain more from preserved cognitive and functional capacity, may ultimately offset the high treatment cost through improved quality of life and long-term productivity.

In contrast, older patients or those with limited life expectancy may derive less benefit from PBT, as they are less likely to experience significant opportunity losses in the post-treatment phase. In such cases, cost-effective photon therapy may be more appropriate. In addition to age, other important considerations include tumor location and biology. Tumors located near critical organs or demonstrating aggressive behavior may justify the use of PBT, while more indolent tumors in less sensitive regions may not.

Future research incorporating biomarkers and patient-specific tumor characteristics may further refine these decisions. As PBT continues to mature and becomes more financially attainable, research should not only explore which tumor types benefit most from this modality, but also which patient populations—based on socioeconomic status, age, and life trajectory—stand to gain the most. This comprehensive approach would empower clinicians to offer more personalized, evidence-based guidance to patients choosing between proton and photon therapies.

Although current research on the benefits of PBT across various cancer types and patient populations remains limited—largely due to its evolving technological landscape, high implementation costs, and restrictive insurance coverage—its clinical potential is undeniable. By carefully evaluating and selecting appropriate patient candidates, the medical community can utilize existing evidence to optimize treatment outcomes and help patients regain the highest possible quality of life following diagnosis. Nevertheless, significant gaps persist. Ongoing and future research is essential to better define the clinical, biological, and socioeconomic contexts in which PBT offers the greatest value. Following the future directions outlined in this review may serve as a useful guide for researchers and policymakers aiming to expand and refine the role of PBT in modern oncology.

## Limitations

During our research, we encountered considerable difficulty in standardizing search results and comparing different RT modalities. Each RT approach offers distinct advantages depending on tissue type, cancer staging, and clinical context, making direct comparisons inherently complex. Furthermore, the limited number of studies involving human subjects significantly restricts the generalizability of current findings. Ethical constraints make it nearly impossible to conduct controlled trials in which patients with identical cancer types and stages receive different RT modalities, and even among such patients, individual variability—such as genetic differences—can lead to markedly divergent responses to therapy. Although some insights were gleaned from available clinical trials, these studies remain relatively scarce.

Another important limitation lies in the heterogeneity of study designs and outcome measures. Some studies focused on inflammatory biomarkers, while others prioritized endpoints such as OS or organ-specific toxicity, making it difficult to draw consistent conclusions regarding the immunologic impact of different modalities. Nevertheless, we have attempted to synthesize these disparate findings to offer a more unified understanding of the immunoinflammatory landscape of concurrent RT.

Additionally, potential publication bias must be acknowledged, as studies reporting positive or novel findings are more likely to be published. A further limitation is the frequent absence of long-term follow-up data, which is critical for assessing chronic inflammatory responses and delayed adverse effects. Lastly, the reliance on preclinical models—while necessary due to practical and ethical constraints—limits the applicability of some results to human physiology, particularly in the case of PBT, which remains under active clinical development. These challenges underscore the urgent need for more standardized, longitudinal, and patient-centered research, despite the considerable logistical and ethical barriers involved.

## Conclusions

This study highlights the evolving landscape of RT and the importance of understanding the immune system’s role in shaping patient outcomes. Our comparison of various modalities—particularly focusing on the inflammatory responses induced by conventional photon-based therapies versus PBT—demonstrates that radiation’s impact extends beyond direct cytotoxicity and involves complex immunological mechanisms.

While acute inflammation may enhance tumor eradication, sustained or dysregulated inflammatory responses can exacerbate tissue damage and compromise patient recovery. Thus, tailoring radiotherapy to strike a balance between efficacy and toxicity is crucial.

This study has three important implications for future research. First, understanding the immune system’s role in radiation-induced responses is crucial for optimizing therapeutic strategies. Second, while early and acute inflammation triggered by radiation can support the eradication of cancer, prolonged inflammation can lead to further patient injury, underscoring the need for a balance in treatment protocols. Third, PBT holds the potential to revolutionize RT in a way similar to how conventional RT did over 130 years ago, marking the beginning of a new era in cancer treatment.

Overall, advancing our understanding of the immunological landscape shaped by radiation modalities may open new doors for synergistic treatment approaches, such as combining immunotherapy with PBT, ultimately enhancing long-term outcomes for cancer patients.

## Abbreviations

3D-CRT: Three-Dimensional Conformal Radiation Therapy
8-OHdG: 8-hydroxy-2-deoxyguanosine
APCs: antigen-presenting cells
CNS: central nervous system
CRP: C-reactive protein
DAMPs: damage-associated molecular patterns
DCs: dendritic cells
DSBs: double-strand breaks
G4 ALC: grade 4 absolute lymphocyte count
GI: gastrointestinal
ICD: immunogenic cell death
ICIs: immune checkpoint inhibitors
IFN: interferon
IGRT: Image-Guided Radiation Therapy
IL-1Ra: interleukin-1 receptor antagonist
IL-1β: interleukin-1 beta
IMRT: Intensity-Modulated Radiation Therapy
IR: ionizing radiation
LET: linear energy transfer
MBD: mean body dosage
MDA: malondialdehyde
MLR: monocyte-to-lymphocyte ratio
NK: natural killer
NLR: neutrophil-to-lymphocyte ratio
OS: overall survival
PBT: Proton Beam Therapy
PFS: progression-free survival
PRRs: pattern recognition receptors
PT: proton radiotherapy
RR: relative risk
RT: radiation therapy
SBPT: stereotactic body proton therapy
SBRT: Stereotactic Body Radiation Therapy
SIII: systemic immune-inflammation index
TGF-β1: transforming growth factor beta 1
TNF-α: tumor necrosis factor-alpha
XRTs: external beam radiation therapies

## Declarations

### Copyright

© The Author(s) 2025.
